# Mesenchymal stem cells in the treatment of osteogenesis imperfecta

**DOI:** 10.1186/s13619-022-00146-3

**Published:** 2023-02-02

**Authors:** Erica Lang, Julie A. Semon

**Affiliations:** Department of Biological Sciences, Missouri University of Science and Technology, 400 W 11th St., Rolla, MO USA

**Keywords:** Osteogenesis imperfecta, Mesenchymal stem cells, Cell therapy, Transplantation

## Abstract

Osteogenesis imperfecta (OI) is a disease caused by mutations in different genes resulting in mild, severe, or lethal forms. With no cure, researchers have investigated the use of cell therapy to correct the underlying molecular defects of OI. Mesenchymal stem cells (MSCs) are of particular interest because of their differentiation capacity, immunomodulatory effects, and their ability to migrate to sites of damage. MSCs can be isolated from different sources, expanded in culture, and have been shown to be safe in numerous clinical applications. This review summarizes the preclinical and clinical studies of MSCs in the treatment of OI. Altogether, the culmination of these studies show that MSCs from different sources: 1) are safe to use in the clinic, 2) migrate to fracture sites and growth sites in bone, 3) engraft in low levels, 4) improve clinical outcome but have a transient effect, 5) have a therapeutic effect most likely due to paracrine mechanisms, and 6) have a reduced therapeutic potential when isolated from patients with OI.

## Background

Osteogenesis imperfecta (OI), also known as brittle bone disease, is a genetic disorder of connective tissue and is identified by bone dysplasia and fragility (Martin and Shapiro 2007; Forlino and Marini 2016). With an estimated 25,000-50,0000 cases in the United States, it affects approximately 1 out of 10,000-20,000 live births worldwide (Palomo et al. 2017; Götherström and Walther-Jallow 2020; Otsuru et al. 2012). OI cases have not been reported to have a gender bias, but some types of OI may be more prevalent in different ethnic groups (Martin and Shapiro 2007).

## Osteogenesis imperfecta

### Epidemiology and clinical manifestation

OI can be mild, severe, or perinatal lethal. The severity depends on the ratio of normal to mutant type I procollagen (Pochampally et al. 2005). Those with mild forms of OI may go through life without a diagnosis, however, severe forms can be diagnosed in utero, at birth, or in early childhood. Diagnosis is made through clinical manifestations, family history, genetic tests, and bone density scans, such as X-rays and DXA/ DEXA scans (Rossi et al. 2019; Mäyränpää et al. 2011). Fractures, bone fragility, skeletal deformity, and short stature are hallmark clinical manifestations of all types of OI (Götherström and Walther-Jallow 2020; Otsuru et al. 2012; Thomas and DiMeglio 2016). Typically, moderate to severe OI presents with multiple fractures after little or no trauma during the prenatal period, at birth, or in early childhood (Forlino and Marini 2016; Götherström and Walther-Jallow 2020). OI patients could have a few fractures over a lifetime or several hundred, dependent on OI severity. Though all bones are at risk, long bones are more commonly fractured. As a systemic disorder of connective tissue, OI symptoms extend beyond the skeletal tissue. OI patients can suffer from ocular complications such as blue sclerae, brittle teeth known as dentinogenesis imperfecta (DI), hearing loss, cerebral hemorrhage caused by birthing trauma, heart disease (valve insufficiency and aneurysms), pulmonary problems (decreased function and repeated infections), and joint laxity (Cheung et al. 2007; Van Dijk and Sillence 2014; Widmann et al. 1999; Etich et al. 2020; Zhytnik et al. 2020). Life expectancy may be shortened for those with severe OI types, but is not affected in mild OI types (Götherström and Walther-Jallow 2020).

### Classification

Initially, OI was categorized into four groups (I-IV) based on clinical features, including fracture severity and rate of occurrence, skeletal deformity, hearing loss, and sclerae color (Thomas and DiMeglio 2016; Sillence et al. 1979). All four categories demonstrated that OI was an autosomal dominant (AD) disorder due to defects in type I collagen (Ralston and Gaston 2019). However, in 2006, a recessive gene was identified for OI, which opened the doors for further identification of additional OI genes (Rossi et al. 2019; Thomas and DiMeglio 2016; Valadares et al. 2014). The original classification system by Sillence *et. al.* was then expanded to 16 groups and was based on the type of genetic mutation (Ralston and Gaston 2019).

Within the expanded Sillence classification, OI symptoms are still inconsistent with severity and morbidity, even in the same classification category. Furthermore, there may be different manifestations and varying disease severities within individuals from the same family (Thomas and DiMeglio 2016; Valadares et al. 2014). Forlino et al., recently proposed a novel classification system based on functional genetics (Table 1) (Forlino and Marini 2016). This method categorizes OI according to gene products that share mechanisms because they function in the same pathway. In this classification, defects in collagen synthesis, structure, or processing are represented by OI group A; defects in collagen modification are included in OI group B; defects in collagen chaperones belong to OI group C; defects in bone mineralization comprise OI group D; and defects in osteoblast development constitute OI group E (Forlino and Marini 2016).

**Table 1:** Overview of OI classification and phenotypes

Forlino Group	Defect	Sillence Type	Gene Symbol	OMIM #	Mode of Inheritance	Severity	Clinical Characteristics
Group A	Collagen synthesis, structure, or processing	I	COL1A1 or COL1A2	166,200	AD	Mild or non-deforming	Triangular face, minimal bone deformity, tinted sclera, fractures before puberty, hearing loss possible
		II	COL1A1 or COL1A2	166,210	AD	Perinatal lethal	Numerous fractures, severe deformity, underdeveloped lungs, collagen improperly formed
		III	COL1A1 or COL1A2	259,420	AD	Severe	Fractures present at birth, tinted sclera, triangular face and spinal curvature, loose joints, poorly developed muscles, aberrant collagen, hearing loss, and respiratory problems
		IV	COL1A1 or COL1A2	166,220	AD	Moderate	Short stature, fractures occur before puberty, brittle teeth and hearing loss possible, sclera normal in color, barrel shaped rib cage, collagen improperly formed
		XIII	SP7/Osterix	614,856	AR	Mild to severe	Joint hyperextensibility
Group B	Collagen modification	VII	CRTAP	610,682	AR	Moderate to severe	Clinically similarly to type IV and II, short stature, short humorous and femur, coxa vara is common
		VIII	LEPRE1	610,915	AR	Moderate to severe	Resembles lethal types II and III in appearance and symptom, normal sclera, deficiency of P3HI, skeletal under mineralization, fractures
		IX	PPIB	259,440	AR	Moderate to severe	Scoliosis, short lower limbs, blue sclera, bowing of limbs, flattened vertebrae, fractures. Similar to types II or III
		XIV	TMEM38B	615,066	AR	Moderate to severe	Multiple fractures, osteopenia, normal dentition, normal sclera, normal hearing
Group C	Collagen chaperones	X	SERPINH1	613,848	AR	Severe	Short limbs, bowing at the thigh, blue sclera, fractures, triangular face, dentinogenesis imperfecta, respiratory distress, bone deforming, multiple fractures, osteopenia
		XI	FKBP10	610,968	AR	Moderate to severe	Progressive malformation, bone fractures, joint contractures, and kyphoscoliosis, no dentinogenesis imperfecta
Group D	Mineralization	V	IFITM5	610,967	AD	Moderate	Clinically similarly to type IV, large calluses at sites of fractures, calcification of membrane between radius and ulna
		VI	SERPINF1	613,982	AR	Moderate to severe	Clinically similar to type III or IV, alkaline phosphatase activity is slightly elevated
Group E	Osteoblast differentiation	XII	SP7	613,849	AR	Mild to moderate	Bowing of extremities, delayed teeth eruption, poor bone mineralization, hyperextensible joints, low bone density, recurrent fractures, osteoporosis, normal hearing, normal sclera
		XV	WNT1	615,220	AR/AD	Moderate to severe	Short stature, low bone density, early onset fractures, vertebral compression and long bone fractures, bluish sclerae, no dentinogenesis imperfecta
		XVI	CREB3L1	616,229	AR	Mild to severe	Prenatal onset of fractures, blue sclerae, bone demineralization, hyperextensibility, decreased ossification of the skull

*Abbreviations*: *AD* Autosomal dominant, *AR* Autosomal recessive

### OI group A

OI group A has defects in collagen synthesis, structure, or processing and includes Sillence types I-IV and XIII (Table 1). Because collagen I is decreased in all these groups, common symptoms of OI group A include bone dysplasia, a bluish discoloration of the sclerae, hyperextensible joints, scoliosis, and easily bruised skin (Marom et al. 2020). In classical OI types I-IV, which represents 85-90% of OI cases, inherited AD mutations occur in genes *COL1A1* and *COL1A2*, which encode proteins used to assemble type I collagen (Palomo et al. 2017; Etich et al. 2020; Valadares et al. 2014). As only one copy of the altered *COL1A1* or *COL1A2* gene is required, these genetic alterations result in a reduced production of quality type I collagen which is essential for the composition of connective tissue (Rauch and Glorieux 2004). The defects can be considered qualitative, which is a structural defect that occurs when an abnormal collagen I molecule is produced, or they can be considered quantitative, which is a haploinsufficiency with type I collagen being structurally normal but synthesized in about half of normal levels (Zhytnik et al. 2019; Lindahl et al. 2015; Marini and Blissett 2013). Maioli et al. showed that 195 mutations, which consisted of nonsense, frameshift, large rearrangement, and splice site mutations, could cause a quantitative defect and are related to milder phenotypes associated with OI type I (Maioli et al. 2019). The same study also showed that 114 mutations, mostly from glycine substitutions, would result in qualitative defects associated with more severe forms such as OI types II-IV. In qualitative defects, the phenotypic severity has been correlated to gene affected, helical location of mutation, and predicted final protein product (Lindahl et al. 2015; Maioli et al. 2019; Marom et al. 2020; Morello and Rauch 2010). However, there remains a great deal of phenotypic variability within each genotype of OI. Thus, it’s difficult to correlate genotype solely based on phenotype, and vice versa; it makes it further difficult to base treatment choice on genotype or phenotype alone.

In OI group A, OI type I is the mildest and classified as non-deforming with persistently blue sclera (Marom et al. 2020). It may involve only a few fractures over a person’s lifetime, with the fracture count decreasing post-puberty. Patients also present with blue sclerae and hearing loss (Marini and Blissett 2013). Due to the mild nature and normal stature of type I OI, it is not always apparent and, therefore, diagnosed. While most cases of OI type I are associated with quantitative mutations (62%), it can be associated with qualitative mutations (25-32%) (Lindahl et al. 2015; Maioli et al. 2019). Interestingly, in a study of 364 OI patients in Italy, clinical differences were not evident between patients carrying qualitative or quantitative defects (Maioli et al. 2019).

Contrary, type II OI is often diagnosed in utero and is typically perinatal lethal, often due to rib fractures and chest wall deformities (Götherström and Walther-Jallow 2020; Marom et al. 2020). Infants who survive the perinatal period typically have shortened extremities, underdeveloped lungs resulting in respiratory complications, malformed ribs and long bones, large soft spots on the top of the skull, and usually don’t survive the first year of life (Marom et al. 2020; Marini and Blissett 2013).

Type III OI is the most severe form of OI among patients surviving infancy. It is classified as severe with progressive malformation of the ribs and long bones, which may result in scoliosis, short stature, restricted mobility, and multiple fractures, especially in the fetal through preschool years (Marom et al. 2020; Sinikumpu et al. 2015). In addition, pulmonary insufficiency and respiratory problems are possible due to the rib cage taking on a barrel shape (Etich et al. 2020). Individuals may also present with triangular facial appearance, hearing impairment, gray or blue sclerae, and DI (Marom et al. 2020; Marini and Blissett 2013). With the current standard of care and early treatment, patients suffering from OI type III may reach satisfactory health beyond the initial years of childhood, though they are often full-time wheelchair users (Sinikumpu et al. 2015; Marini and Dang 2000).

Type IV OI demonstrates clinical variability and is considered moderately severe (Marini and Dang 2000). Fractures are more common before puberty or post middle age, with malformation of the bones varying from mild to severe. Similar to other groups, these patients may exhibit spinal curvature, hearing impairment, DI, a triangular-shaped face, barrel shaped rib cages, and osteoporosis (Marom et al. 2020; Marini and Dang 2000).

Type XIII OI is the only group to have an autosomal recessive (AR) mutation, and the only one to have a mutation in a gene other than type I collagen. It’s mutation in the *SP7/Osterix* gene results in a phenotype similar to OI type IV (Marini and Dang 2000). Because it ranges from mild to severe, this group displays common OI symptoms, including joint hyperextensibility, bowing of upper and lower limbs, and Wormian bones (Morello and Rauch 2010; Marini and Dang 2000). Additionally, this group may exhibit mild scoliosis and osteoporosis while not presenting DI or blue sclera (Marini and Dang 2000).

### OI group B

OI group B, which includes Sillence types VII, VIII, IX, and XIV, are all AR and result from mutations leading to post-translational modification of collagen (Thomas and DiMeglio 2016; Sillence et al. 1979). Type VII OI, a qualitative defect, is caused by mutations in the CRTAP gene, which is responsible for collagen prolyl 3- hydroxylation (Martin and Shapiro 2007; Morello and Rauch 2010; Ward et al. 2002). The mutated form of CRTAP leads to moderate symptoms, while the absence of CRTAP results in lethality (Etich et al. 2020). Clinical manifestations of the mutated form include fractures at birth, bluish sclerae, coxa vara, and a short humerus, femur, and stature (Etich et al. 2020; Ward et al. 2002; Srisaarn et al. 2019; Roberts et al. 2018). Type VIII is caused by a mutation in the LEPRE1 gene resulting in a deficiency of prolyl 3-hydroxylase 1 (P3H1) (Otsuru et al. 2012; Morello and Rauch 2010). It can be lethal or severely deforming with a lack of mineralization and a growth deficiency, thus resembling Sillence types II or III in appearance and symptoms (Otsuru et al. 2012). Type IX is caused by a mutation in the PPIB gene, which is responsible for encoding CRTAP (mentioned above) (Thomas and DiMeglio 2016; Harrington et al. 2014; Womack 2014). These patients experience severe growth deficiency as well as shortened and bowed limbs (Harrington et al. 2014; Womack 2014). Type XIV is an AR mutation in the TMEM38B gene, which encodes TRIC-B, an ion channel responsible for maintaining intracellular calcium release. When this ion channel is disrupted, proband type I collagen synthesis is dysregulated. Patients with type XIV OI have blue sclera, osteoporosis, bowed limbs, and long slender bones (Lv et al. 2016).

### OI group C

OI group C results from chaperon defects and includes Sillence types X and XI, which are both inherited in an AR manner (Cheung et al. 2007; Harrington et al. 2014). There is only one reported case of Type X OI in a human (Womack 2014). Genetic testing found a mutation in SERPINH1 that encodes for HSP47, which aids in the function of collagen trafficking (Womack 2014). HSP47 was found to monitor the integrity of type I procollagen’s helix and monitor it’s travel from the endoplasmic reticulum to the golgi. The parents were reported as healthy, but the three-year-old boy had severe OI and was born with a triangular face, blue sclerae, short limbs with bowing at the sides, and fractures. As he aged, he developed renal stones, a subdural hematoma due to trauma, and chronic lung disease (Womack 2014). These respiratory complications are believed to be a result from scoliosis, multiple fractures to the ribs, and altered chest construction, which the boy experienced (Thiele et al. 2012). OI type XI is also uncommon and is limited to a few individual cases. It is a mutation in the FKBPP10 gene, which encodes the FKBP65 protein found in the endoplasmic reticulum. Additionally, this gene is also a known cause for Bruck syndrome, an AR disorder also distinguished by bone fragility (Yüksel Ülker et al. 2021). Type XI includes symptoms of progressive malformation, bone fractures, joint contractures, and kyphoscoliosis (Womack 2014).

### OI group D

OI group D has defects in bone mineralization and includes Sillence types V and VI. Type V OI is inherited in an AD manner and is caused by a mutation that produces a new start codon for the IFITM5 gene resulting in abnormal collagen production due to atypical mineralization (Palomo et al. 2017; Rossi et al. 2019; Shapiro et al. 2013). These patients develop hyperplastic calluses (HPCs) after injury, which begins as soft tissue formations consisting of loose collagenous networks (Cheung et al. 2007; Shapiro et al. 2013). As time goes on, the callus evolves defined boundaries and eventually ossifies, with the innermost region of the callus showing trabeculae of woven bone (Shapiro et al. 2013; Hilton 1953; Brenner et al. 1989). Cortical bone appears mesh-like under the microscope and lamellae are irregularly located. In addition to this, interosseous membrane calcification occurs between the radius and ulna as well as the tibia and fibula (Palomo et al. 2017; Grover et al. 2013). This results in the difficulty of supination/pronation movements. Type VI OI, inherited in an AR manner, is caused by a homozygous mutation in the SERPINF1 gene (Rossi et al. 2019; Valadares et al. 2014; Homan et al. 2011). Histology of the cortical bone depicts a fish scale appearance of the lamellae due to a deficiency of bone mineralization (Ralston and Gaston 2019). Patients with type VI OI clinically resemble type IV OI with moderate severity, typically no fractures at birth, and tinted sclerae during infancy (Rossi et al. 2019).

### OI group E

OI group E, including Sillence types XII, XV, and XVI, has defects in osteoblast development. In type XII, there is a frameshift mutation in SP7 which causes a premature stop codon. This premature stop codon produces abnormal osterix, which is a transcription factor for osteoblast differentiation (Martin and Shapiro 2007). In one clinical case, fractures were noted at 3 months of age and teeth eruption and motor milestones were delayed (Kang et al. 2017). Additionally, he experienced bowing of the upper and lower limbs, mild asymmetry of the face, and hyperextensible joints. His hearing was unaffected, sclerae presented as white, and he fell within the normal weight range of his age (Kang et al. 2017). Type XV OI is caused by a mutation to WNT1 gene, in turn causing failed activation of β-catenin signaling. This signaling pathway has a role in bone formation and osteoblast function. Patients with type XV OI present early onset fractures, bluish sclerae, a reduction in bone density, and short stature. However, similar to type XII OI, hearing and tooth development were noted as normal (Kang et al. 2017; Symoens et al. 2013). In type XVI OI, there is a CREB3L1 deletion (Symoens et al. 2013). This gene is known for encoding an endoplasmic reticulum stress transducer known as OASIS, which regulates type I procollagen and osteoblast differentiation. A Turkish family suffering from type XVI presented with a nine-month-old that passed away from severe symptoms that included fractures, wide fontanelles, beaded ribs, callus formation, and pulmonary infections. The second pregnancy was terminated at 19 weeks gestation. Upon post-mortem examination, they discovered multiple fractures, bowed humerus and femora, and extremely thin ribs. The parents later gave birth to a healthy daughter who only exhibited blue sclerae (Symoens et al. 2013).

### Treatment

There is no cure for OI, and techniques to manage the disease do not address the underlying molecular pathology. Instead, treatment and management strategies focus on providing symptomatic relief and maximizing bone health by minimizing bone deformities, pain, and the morbidity of fractures. This is usually accomplished through multiple approaches, including physical therapy, surgical treatments, vitamin supplements, pharmaceuticals, and treatment of other complications (Rossi et al. 2019; Widmann et al. 1999; Etich et al. 2020). The goal of physical therapy and habilitation is to strengthen the muscles and to retain and improve mobility. It will vary depending on the severity of OI and may include occupational therapy, hydrotherapy, and weight-bearing physical activity. Surgical treatment may be required for more severe OI cases and are crucial for fixing fractures and correcting limb deformities (Forlino and Marini 2000; Hidalgo Perea and Green 2021; Laron and Pandya 2013; Marr et al. 2017). This can entail placing rods in the long bones to correct bone deformities and reduce fractures. Spinal fusion, which connects vertebrae, may also be used. Vitamin supplements, specifically Vitamin D and calcium, are used for therapy for both children and adults with all levels of severity and types of OI (Götherström and Walther-Jallow 2020; Thomas and DiMeglio 2016).

The current gold standard pharmaceutical agents to treat OI are bisphosphonates (Zhytnik et al. 2020; Drake et al. 2008; Dwan et al. 2016). Currently, they are widely used to treat moderate to severe forms of OI and are safe enough to use in children (Otsuru et al. 2012; Thomas and DiMeglio 2016). Patients take bisphosphonates orally or intravenously to increase bone mass (Drake et al. 2008; Dwan et al. 2016). Bisphosphonates increase bone mineral density by inactivating osteoclasts, thus reducing bone resorption (Drake et al. 2008; Dwan et al. 2016). Though bisphosphonates have been shown to increase overall bone mineral density, it is uncertain if it improves bone strength or alleviates the risk of bone fractures, improves functional mobility, reduces pain, or improves growth (Drake et al. 2008; Dwan et al. 2016; Chan and Götherström 2014). Additionally, bisphosphonates have been shown to negatively affect other biomechanical properties of bone (Mashiba et al. 2000). Other anti-resorption pharmaceuticals and osteoclasts inhibitors are being developed, including the RANKL antibody denosumab (Bargman et al. 2012; Semler et al. 2012). Growth hormone and parathyroid hormone are also being explored both independently and in combination with other treatments, such as bisphosphonates (Antoniazzi et al. 1996; Antoniazzi et al. 2010; Orwoll et al. 2014).

As mentioned, the current treatments mainly manage the disease and do not correct the underlying molecular defect. In order to habilitate those with OI, gene and cell therapy strategies have been explored. A successful genetic treatment for OI, a model disorder for dominant negative defects of structural proteins, will eliminate the dominant negative mutant allele and degrade abnormal COL1A1/2 transcripts (Chan and Götherström 2014; Chamberlain et al. 2004). A successful cellular treatment for OI will safely provide exogenous cells, ideally early on in human development, that will decrease the severity of the disorder by contributing to bone formation (Jones et al. 2014). Cellular therapy for OI, sometimes used in conjunction with gene therapy, has been explored for over 20 years (Chan and Götherström 2014; Caplan 1995; Ramesh et al. 2021). One cell type specifically has shown promise in reducing the number of fractures over a lifetime, reducing surgical and physical habilitation, and even correcting underlying molecular defect: mesenchymal stem cells (MSCs).

## Mesenchymal stem cells

### Introduction

MSCs, also referred to as medicinal signaling cells, mesenchymal stromal cells, and marrow stromal cells, have been shown to be safe and effective for cell therapy in numerous applications, including skeletal disorders (Wei et al. 2013; Arthur and Gronthos 2020). First isolated 50 years ago from the adherent portion of bone marrow, they are a heterogeneous cell population that can adhere to plastic and maintain a fibroblast-like morphology (Squillaro et al. 2016; Saeed et al. 2016). Per the International Society for Stem Cell Research (ISSCR) and the International Society for Cell Therapy (ISCT), MSCs must express surface antigens CD73, CD90, and CD105 and must have low to no expression of CD14, CD34, CD45, and CD79 (Pittenger et al. 2019; Dominici et al. 2006). Per these criteria, they must also differentiate into bone, fat, and cartilage in vitro.

Originally identified in adult bone marrow, MSCs are now sourced from additional tissues, including peripheral blood, umbilical cord tissue and blood, dermal tissue, adipose tissue, and gingival tissue (Götherström and Walther-Jallow 2020; Guillot et al. 2008b; Galipeau and Sensébé 2018; Undale et al. 2009). Bone marrow derived MSCs (BMSCs), still the most common source of MSCs for clinical trials, are found along the endosteum and are isolated by aspiration and separation with a ficoll gradient (Baghaei et al. 2017). However, the bone marrow aspirate that BMSCs are isolated from is an invasive harvest, especially in patients with skeletal disorders such as OI (Galipeau and Sensébé 2018; Undale et al. 2009). Consequently, autologous BMSCs used to treat OI require gene therapy. Dental pulp MSCs are mainly sourced from extracted wisdom teeth that are enzymatically digested and are a less invasive harvest than BMSCs. Though they have been reported to have a higher proliferative rate than BMSCs, only a low number of cells are available for isolation due to the small size of the dental pulp (Ponnaiyan and Jegadeesan 2014). Adipose derived MSCs (ASCs) are extracted from subcutaneous fat and isolated from the stromal vascular fraction (SVF). They have similar therapeutic effects of BMSCs, are acquired by less invasive means than BMSCs, and provide a large number of cells after isolation (Gonzalez-Rey et al. 2010; Dykstra et al. 2017; Levi and Longaker 2011; Mazini et al. 2019). Using ultrasound guidance, cardiocentesis has been used to collect MSCs from peripheral blood from a first trimester fetus during pregnancy (Guillot et al. 2008a; Vanleene et al. 2011). These fetal MSCs (fMSCs) were found to be more primitive and immunologically naïve (Mäyränpää et al. 2011; Mazini et al. 2019) than BMSCs, though they are collected in low numbers (Götherström and Walther-Jallow 2020; Jones et al. 2014; Németh et al. 2009). Umbilical cord MSCs are taken from the umbilical cord and typically hold little ethical or political ramifications due to their classification as medical waste (Jones et al. 2014; Rady et al. 2020). Similar to ASCs, they are less invasive to acquire than BMSCs and can provide a large number of MSCs (Jones et al. 2014; Rady et al. 2020; Salehinejad et al. 2020; Amati et al. 2017; Ballen et al. 2013; Ren et al. 2021). Placenta-derived MSCs, also called early chorionic stem cells (CSC), can be isolated during ongoing pregnancy without harm to the fetus, and, therefore, used without ethical restrictions (Jones et al. 2014). These cells can be isolated from various parts of placental tissue including chorionic villi, deciduae, amniotic membrane, and chorionic membrane using different methods (Yi et al. 2020). These cells can be harvested in high numbers and proliferate at higher rates in vitro, reducing the amount of time in culture. MSCs obtained from a different source, fetal livers (FL-MSCs), recently have increased interest due to their higher proliferative rates, increased differentiation capacity, and long-term immunomodulatory properties (Yu et al. 2021). Similar to CSCs, the faster growth kinetics of FL-MSCs reduces the amount of time in culture.

Though each source of MSCs has limitations and advantages, MSCs overall have a versatile nature in the clinic. They can be directly injected for cell therapy, or they can be combined with a biomaterial(s) to develop tissue engineered constructs (Kangari et al. 2020; Vadalà et al. 2016; Gao et al. 2016; Kean et al. 2013). They can be used as-is, mixed with other cell types, or be sorted out via a cell surface marker to make a more homogenous cell population (Zhang et al. 2015; Parekkadan and Milwid 2010). They can come from autologous, allogeneic, or mixed sources. They can be used in an unaltered, naïve state; they can be primed down a desired lineage; or they can be modified through the addition of an external gene (Kangari et al. 2020; Mount et al. 2015). Recently, just their exosomes have been used, which can also be cryopreserved and used off-the-shelf (Zhang et al. 2015; Otsuru et al. 2018).

### Mechanisms of actions

Originally, MSCs were of clinical interest because they could provide a continuous source of cells, and they were multipotent, differentiating into osteoblasts, chondrocytes, and adipocytes (Wei et al. 2013). However, the current paradigm is that MSCs are useful in clinical applications because they are generally considered non-immunogenic, possess immune modulatory properties, reduce sepsis, and secrete an extensive array of factors that act on endogenous cells (Németh et al. 2009; Rady et al. 2020; Aggarwal and Pittenger 2005; Le Blanc 2003).

Despite the therapeutic promise of MSCs, there have been many inconsistencies in clinical trials in regard to their therapeutic efficacy (Ramesh et al. 2021). Additionally, pre-clinical results have shown limitations that must be addressed to provide more clinical success for all types of MSCs. For example, there are several reports showing that MSCs are directly or indirectly involved with cancer (Patel et al. 2010; Strong et al. 2012; Lazennec and Jorgensen 2008). BMSCs have also been shown to worsen bacterial infection, resulting from their anti-inflammatory nature Saeed et al. 2016; Meisel et al. 2011). Regardless of the source, MSCs senesce and deteriorate under standard culture conditions, as well as with the increasing age and poor pathology of MSC donor (Jones et al. 2014; Gonzalez-Rey et al. 2010; Siegel et al. 2013; Choudhery et al. 2014; Scruggs et al. 2013; Pandey et al. 2011). In some cases, the administration of cryopreserved MSCs, which are thawed at the bedside, have shown limited therapeutic effects (Otsuru et al. 2018; Moll et al. 2014; François et al. 2012). If cryopreserved MSCs must be thawed and expanded in culture, it limits their use to hospitals with Good Manufacturing Practice (GMP) facilities (Otsuru et al. 2018; Moll et al. 2014; François et al. 2012). With only ~two dozen GMP facilities in the U.S., it leaves about half the number of states without these abilities (Phinney and Galipeau 2019). MSCs from different sources appear to vary in secretion factors, differentiation capacity, and homing abilities. For example, MSCs from fetal tissue are considered more primitive, have longer telomeres, and a higher proliferative capability (Chan and Götherström 2014; Saeed et al. 2016; Musiał-Wysocka et al. 2019). In addition to this, their yield, differentiation capabilities, and growth kinetics vary significantly between individuals (Jones et al. 2014; Ramesh et al. 2021; Phinney et al. 1999). Different donors have provided MSCs with distinct genomes, different phenotypes, and varying levels of therapeutic efficacy (Phinney et al. 1999; Siddappa et al. 2007; Zhou et al. 2008; Zhukareva et al. 2010). The variation in the therapeutic efficiency of different donors causes difficulties with interpreting clinical outcomes.

### MSC alterations in patients with OI

Almost all of the preclinical and clinical trials for cell therapy in OI used allogenic cells, as getting biopsies from patients with systemic conditions is not favorable. Another limitation to using autologous cells is that, similar to other diseases, MSCs from OI patients have been shown to be less therapeutic (Chamberlain et al. 2004; Kaneto et al. 2017).

Murine models of OI have demonstrated that MSCs from both bone and adipose tissue have a reduced osteogenic potential. While Pereira et al. showed no difference in growth rates between BMSCs from OI mice and wild-type (WT) mice, the alkaline phosphatase (ALP) activity and Alizarin red stain in BMSC cultures at 2 weeks was drastically reduced in BMSCs cultures from OI mice (Pereira et al. 1998). Similarly, Liu et al. showed that ASCs from OI mice had reduced ALP and collagen levels due to decreased YAP signaling, although they also reported a decrease in colony forming units (CFUs) from ASCs derived from OI mice. They reported that autologous ASCs may be able to divide and differentiate into osteoblasts, however, they will be defective cells that produce minimal or aberrant collagen (Liu et al. 2021).

Similar results were shown in MSCs derived from OI patients. During characterization, BMSCs from OI patients had similar surface marker profiles to BMSCs from patients with normal skeletal development (Kaneto et al. 2017). However, BMSCs from OI patients had a reduced expression of osteogenic markers during osteogenic differentiation, a decreased commitment to osteogenic lineage, and a higher propensity for adipogenesis over osteogenesis (Kaneto et al. 2017). Furthermore, BMSCs from OI patients have point mutations in *COL1A1* or *COL1A2*, worse collagen processing, worse collagen stability, and worse collagen structure (Chamberlain et al. 2004).

ASCs derived from OI patients also demonstrated a reduced therapeutic potential. Abuhantash et al., took ASCs from OI patients, differentiated them into osteoblasts, and compared their extracellular vesicles (EVs) to those of osteoblasts from healthy patients (Abuhantash et al. 2020). The EVs from OI cells had the persistent expression of fibronectin, Fibulin-1 and -2, and laminas, which indicates an immature extracellular matrix (ECM). However, an organized and mature ECM is required for proper ECM mineralization. This study also showed that annexins, which are critical for ECM mineralization due to their calcium binding, were down regulated in EVs from OI cells.

As both BMSCs and ASCs derived from OI mice and patients have shown a reduced osteogenic potential and an impaired mineralization ability, it suggests these cells may contribute to the bone fragility in OI patients. This is an essential argument for restricting the use of autologous cells in OI patients. If autologous cells were to be used, they would always require gene therapy to correct their mutation.

## MSCs in pre-clinical studies

Most of the pre-clinical models of OI consist of transplanting murine or human MSCs into mice (Table 2). Success criteria for these models is typically a reduction in the number of fractures as well as an increase in bone stiffness compared to PBS-treated controls. In addition to evaluating the therapeutic efficacy of cell therapy, these models can serve as a tool to examine the molecular and cellular mechanisms of MSC treatments. Murine models include transgenic, naturally occurring mutations, knock-outs, and knock-ins (Forlino et al. 1999; Daley et al. 2010).

**Table 2:** Preclinical studies of MSCs in the treatment of OI

Cell Type	Model	Type of OI	Methods	Results	Reference
**Murine Cells or Cell Products into Mice**					
Allogeneic BMSCs from WT mice	Transgenic mice, 3 week old, irradiated	I	IP	• Low engraftment @ 1 mo • Differentiate into fibroblasts in multiple tissues @ 2.5 mo • Continual source of new cells • Only small effects on bone phenotype @ 1 mo • Increased collagen and mineral content @ 1 mo	Semler et al. 2012
Allogeneic BMSCs from WT mice	*oim* mice, 8-10 week old, irradiated	III	IO	• Robust engraftment @ 1 mo • Long term engraftment @ 3 and 6 mo • Differentiation into osteoblasts @ 3 mo • Continual source of new cells @ 6 mo • Improved cortical structure and strength @ 3 and 6 mo	Strube et al. 2009
Allogeneic BMSCs from WT mice	BrtIIV mice, E13.5-14.5	III/IV	IUT	• Eliminated perinatal lethality **All results measured @ 2 mo of age** • Low engraftment • Differentiated into functional osteoblasts • Improved bone mechanics • Improved mineralization and cortical thickness	Théry et al. 2009
Genetically modified ASCs from WT mice	COL1A1 knockout, 8 week old males	I	IV	**All results measured @ 4 weeks after treatment** • Genetically modified ASCs migrated to femur • Genetically modified ASCs differentiated into bone cells • ASCs improved cortical structure and thickness Further improvements when combined with Nell1 Most improvement when genetically modified with NELL1	Thiele et al. 2012
ASCs from WT mice, OI mice, and OI mice with genetic modification	COL1A1 knockout, 8 week old males	I	IO	**All results measured @ 4 weeks after treatment** • Cells migrated to femur • Cells differentiated into osteoblasts • Promoted bone formation • ASCs from WT mice improved bone structure, thickness, and mechanical properties • Genetically modified ASCs from OI mice improved all above but not as much • ASCs from OI mice were not therapeutic	Shapiro et al. 2013
BMSC-derived EVs from WT mice	G610C knock in, 3 weeks old	I of IV	IV once / week, for 4 weeks	**All results measured @ 2 weeks after last treatment** **(@ 2 mo of age)** • Increased bone growth • miRNA depletion in EVs removed therapeutic effects	Pereira et al. 1995
**Human Cells into Mice**					
Human fMSCs, 10 week old fetus 1 donor	*oim* mice, E13.5-E15	III	IUT	• Donor cells persisted in numerous tissues @ 3 mo of age More cells found at 1 week of age than 3 mo of age Retention was greater in bone (5% engraftment) • Donor cells accumulated in areas of active bone formation, remodeling, and fracture sites • Remained as progenitors in bone marrow but differentiated into osteoblasts in bone • Improved bone mechanics @ 1, 2, and 3 mo after birth • Reduced fractures @ 1, 2, and 3 mo after birth	Mäyränpää et al. 2011
Human fMSCs, 10 week old fetus 1 donor	*oim* mice, E13.5-E15	III	IUT	**All results measured @ 2 mo of age** • Significant reduction in femoral fractures • Donor cells engrafted in bone (5%) • Differentiated into functional osteoblasts, expressed osteocalcin • Increased matrix stiffness • No changes in bone morphology	Mazini et al. 2019
Human fMSCs, 10-12 week old fetus, primed with SDF-1	*oim* mice, 2-3 day old neonates	III	IP	**All results measured @ 2 mo of age, compared to unprimed cells** • Increased migration to bone and bone marrow • Higher engraftment in bone and bone marrow • More therapeutic benefit • Reduced fractures	Valadares et al. 2014
Human CSC, 9-10 week old fetus	*oim* mice, 2-3 day old neonates	III	IP	**All results measured @ 2 mo of age** • Less fractures • Increased bone ductility and volume • did not affect bone length or cortical bone formation • Decreased bone brittleness • Differentiated into functional osteoblasts • Upregulated endogenous genes for ossifications • Homed to epiphysis of long bones	L. et al. 2019
Genetically modified BMSCs from OI patient	NOD/SCID, 2-3 month old	NA	SD	**All results measured @ 2 mo of age** • In vivo bone formation • Increased collagen processing, stability, and structure	Khillan et al. 1991

*Abbreviations*: *mo* month/months, *EVs* Extracellular vesicles, *IO* Intraosseous, *IV* Intravenous, *IP* Intraperitoneal, *IUT* In utero transplantation, *SD* Sub-dermal transplant, *NA* Not addressed, *WT* Wild type

The development of early OI models was based on mutations in the proα1(I) chain of type I collagen, representing moderate-to-severe OI types. A COL1A1 knockout can either be homozygous or heterozygous. The homozygous models are lethal, as they produce no type I collagen. The heterozygous models represent moderate to severe OI, as they produce reduced amounts of normal type I collagen. The human minigene model is another early model and is based on 41 missing exons in the central region of the COL1A1 gene. The shortened human minigene associates with normal murine genes, which reduces the amount of normal type I collagen produced (Khillan et al. 1991; Pereira et al. 1993; Pereira et al. 1995). The BrtIIV mouse model is a dominant model of OI that shows biochemical and phenotypic features reflective of moderate-to-severe human type III/IV OI (Forlino et al. 1999; Gioia et al. 2012). This model provides offspring with phenotypic variation in bone strength, number of fractures, and skeletal deformities. Mice can display perinatal lethality or long term survival capable of reproduction. The *oim* mouse is the only naturally occurring OI mouse model. It is a recessive mouse model with a rare mutation in the pro-α2(I) chain (Chipman et al. 1993). With progressive deformities and skeletal fractures, it reflects human type III OI. The G610C is a knock-in mouse model with a glycine substitution mimicking the G-to-T transversion at nucleotide 2098 found in the Old Order Amish (OOA) kindred of Lancaster County, Pennsylvania (Daley et al. 2010). Therefore, the mice have the same phenotypic variation as the OOA kindred, with mild to moderate disease, reduced body mass, increased fractures, and decreased bone strength (Daley et al. 2010).

In our literature search of preclinical in vivo studies of MSC treatment for OI, we found that the most common mouse model used was the *oim* mouse (45% of papers). While 54% of the papers evaluated murine MSCs, 45% evaluated human MSCs. MSCs sources included BMSCs (45% of studies), fMSCs (27%), ASCs (18%), and CSC (9%). Cells were administered both pre- and postnatally, and were injected via i.v., i.p., IO, IUT.

### Murine MSCs

In an early study by Pereira et al., BMSCs were extracted from WT mice and injected i.p. into 3-week old irradiated OI mice expressing the human mini gene for COL1A1 (Pereira et al. 1998). DNA from the donor cells were found in multiple tissues, with no statistical differences in engraftment levels at 1 or 2.5 months after the infusion. Engraftment levels were low and did require a high dose of radiation. However, the donor cells did account for a significant percentage of fibroblasts suggesting that the BMSCs were a continuous source of cells. The effects on bone phenotype were minimal at 1 month post infusion. However, bone mineralization did increase by a small but significant amount, making them less prone to breakage.

In order to overcome the low engraftment of BMSCs, Sinder et al., transplanted BMSCs from WT mice directly into the bone marrow of *oim* mice (Sinder et al. 2020). This method of cell transplantation did appear to increase engraftment of BMSCs, with donor cells accounting for ~ 19% of the cells found on the cortical endosteal surface at 1 month post-transplantation. However, it must be noted that these robust results were limited to mice with a high dose of radiation, as engraftment remained low in nonirradiated mice. The engrafted cells were found 6 months after treatment, concentrated in specific regions of bones. The engrafted cells were shown to have differentiated into osteoblasts, suggesting BMSCs transplanted directly into the bone could serve as a continued source of osteoblast progenitors. In addition to improved cellular outcome, the cortical structure and mechanical properties of recipient bone were improved 3 months after treatment. As OI has been shown to only require 40-75% of osteoblasts to secrete aberrant collagen, directly injecting BMSCs into bone may provide functional cells in enough numbers to overcome the clinical manifestation of OI (Cabral and Marini 2004).

A limitation in the above two studies is the need to use a high dose radiation in order for cells to engraft. To overcome this, Panaroni et al., transplanted cells during the prenatal period when there is rapid skeletal development and potentially spontaneous fractures occurring (Panaroni et al. 2009). When BMSCs from WT type were transplanted intrauterine into BrtIIV mice, perinatal death was avoided, and a higher percentage of offspring survived. The donor cells engrafted into both hematopoietic tissue and nonhematopoietic tissues, though at low levels. The cells that were found engrafted in bone were found in clusters, similar to the results from BMSCs that were directly injected into bone (Sinder et al. 2020). They differentiated into trabecular and cortical osteoblasts which produced 20% of type I collagen in the mice. This suggests that a relatively small population of donor cells can provide a significant amount of normal collagen. Additionally, there were marked improvements in the bone’s mechanical properties/geometry and the mineralization content at 2 months after birth.

Liu et al., preferred to investigate ASCs because they are easy to acquire, have similar differentiation properties as BMSCs, and are available in large numbers (Liu et al. 2020). Though they showed that ASCs systemically administered into COL1A1 knockout mice improved cortical structure and thickness, they had more robust results with ASCs that were co-administered with Nell1, a pro-osteogenic factor shown to promote bone formation. The results were improved even further when ASCs were transduced with a lentivirus containing the NELL1 gene. The genetically modified ASCs could be systemically administered and migrate to the femur, where they were able to differentiate into bone cells. Though the ASCs showed improved femoral microstructure and promoted bone formation, the results were more so with NELL1 or Nell1. They did have limited improvement in femur performance, which may be due to a single dose of genetically modified ASCs or a low number of ASCs injected. Engraftment levels were not reported. The genetically modified ASCs had slightly better efficacy than administrating ASCs with Nell1 protein.

To increase engraftment and create longer lasting effects, different groups have tried to develop strategies to isolate MSCs from OI patients, genetically modify them to correct the mutation, and then return the cells to the same patient. Another study by Liu et al. tested if cells from OI mice could be genetically corrected and, therefore, therapeutically effective. They isolated ASCs from OI mice and transfected them with COL1A1 (Liu et al. 2021). The genetically modified ASCs were able to produce normal type I collagen and were able to differentiate into bone-lineage cells in vitro before re-transplantation to mice. They then injected the modified ASCs into the bone marrow of 8 week old mice and evaluated them 4 weeks later. They significantly improved the microarchitecture, improved mechanical properties, and promoted bone formation. Despite this success, genetically modified strategies have a limitation when used in vivo. The neomycin resistant gene used for transfection can be a target of the immune system for destruction. This is one of the biggest hurdles to evaluating genetically modified MSCs in the clinic.

### Human MSCs

There is a high level of homology between the Col1α2 protein in humans and mice, as well as other proteins involved in bone formation. Therefore, many groups have evaluated human MSCs in a mouse model. In another study designed to test the efficacy of genetically modified cells from OI individuals, Chamberlain et al. isolated BMSCs from two OI patients and used an adeno-associated virus to insert a construct designed to inactive mutated *COL1A1* alleles (Chamberlain et al. 2004). They showed the defect was corrected in vitro. Though they didn’t test it in an OI model, they did evaluate bone formation in the skin of NOD/SCID mice. In addition to showing bone formation in vivo, the genetically modified human BMSCs from an OI patient showed improved collagen processing, stability, and structure.

Instead of using adult cells for therapy, Guillot et al., examined a fetal to fetal approach (Guillot et al. 2008a). The rationale for in utero transplantation (IUT) is in agreement with Panaroni et al., discussed above (Panaroni et al. 2009). Guillot et al., used MSCs derived from first trimester fetal blood (fMSCs), as they are less linage-committed, faster growing, and less immunogenic than adult MSCs. To determine if these primitive MSCs could be effective in preventing and treating OI, Guillot et al., transplanted human fMSCs in utero into *oim* mice (Guillot et al. 2008a). Donor cells were found to engraft in various sites including, heart, lung, brain, and up to 5% in skeletal tissues up to 8 weeks after birth. The human donor cells tended to gather around healing fracture sites as well as bone areas with active formation, similar to Panaroni and Sinder’s results using BMSCs from WT mice. The injected cells were shown to have differentiated into osteoblast, and bone mechanics, length, and thickness were improved. Fractures were drastically reduced.

Another study further explored IUT of human fMSCs into *oim* mice (Vanleene et al. 2011). Again, fractures were drastically reduced, despite low engraftment levels (< 5%) measured at 8 weeks. This study showed that osteoblasts derived from donor cells secreted normal collagen, which decreased hydroxyproline content in the bone, creating more carbonated, or a more mature, apatite crystal structure. Thus, the bone matrix was more stiff, reducing bone brittleness. This coincides with Panaroni’s IUT of BMSCs that showed improvement in crystal homogeneity (Panaroni et al. 2009). These studies suggest that the decrease in fracture rate from MSC treatment may be due to a change in bone tissue properties, specifically mineralization, rather than a change in bone shape or size. An interesting note about this study was that the investigators found a gender difference with female mice showing more mature apatite crystals. Though their objective was not to evaluate gender differences, it does correlate with previous studies showing that gender differences can affect osteogenic potential of donor cells, proliferation and survival of donor cells, and bone healing (Hong et al. 2009; Corsi et al. 2007; Strube et al. 2009). This is a meaningful reminder to report the gender of the mice as well as the gender of the donor in literature.

Jones et al. also attempted to overcome the low engraftment in bone and, therefore, the transient effects of MSCs (Jones et al. 2012). They primed fMSCs with stromal cell derived factor 1 (SDF-1), a chemokine that upregulates CXCR4. CXCR4 increases cell migration towards bone and bone marrow. Oim neonates at 2-3 days were injected i.p. At 8-weeks post-transplantation, primed fMSCs had higher engraftment, drastically reduced fractures compared to their unprimed counterparts. The decrese in bone brittleness was associated with the higher engfatment levels of fMSCs.

Though fMSCs show great promise, they are limited by their low numbers and availability. Another primitive MSC used in a pre-clinical model of OI was placenta-derived MSCs (CSC) (Jones et al. 2014). Jones et al. administered CSCs i.p. into neonate *oim* mice. At 8 weeks post-transplant, there were reduced fractures and increased bone flexibility. Bone volume was increased, though again, the length of the bone was unaffected. Similar to other studies, donor cells engrafted preferentially to the growth plate and fracture sites (Sinder et al. 2020; Panaroni et al. 2009; Guillot et al. 2008a). Donor cells differentiated into osteoblasts that produced normal collagen. They also showed that donor cells increasing hypertrophic chondrocytes, indicating fMSCs transplantation may increase endogenous endochondral ossification. Again, fractures were reduced.

### Extracellular vesicles

As previously mentioned, there are limitations with MSCs, regardless of the source. To get around the limitations of MSCs, investigators are still working to find the ideal source of MSCs, the optimal method to culture cells, and new techniques to deliver the cells. Of more and more interest is using extracellular vesicles (EVs), rather than the entire cell. EVs, including exosomes and microvesicles, contain proteins, RNAs, and miRNAs (Otsuru et al. 2018; L. et al. 2019). EVs derived from different sources of MSCs have been shown to be therapeutically effective in the treatment of several diseases (Otsuru et al. 2018; Katsuda et al. 2013; Phinney and Pittenger 2017; Phinney et al. 2015; Théry et al. 2009).

Otsuru et al. isolated EVs from both human and murine BMSCs (Otsuru et al. 2018). They infused EVs into OI mice at 3 weeks and then once a week for a total of four treatments. They compared EVs between species, to parental MSC counterpart, and all groups to PBS injected controls. Two weeks after the last injection, bone growth was evaluated. EV-treated groups had similar measurements as BMSC-infused groups, with some bones increasing in length while others did not. EV therapy increased the proliferation of chondrocytes in the growth plate, increasing bone growth. Looking for a mechanism, they narrowed it down to the miRNA(s) transported within the EVs, though it is unclear which miRNA provided this therapeutic effect. These EVs can be stored frozen, freshly thawed and directly infused into patients, and have less risk for venous thrombosis and pulmonary embolism.

## MSCs in clinical studies

### BMSCs

In a foundational clinical trial, Horwitz et al., wanted to see if allografts would be accepted by OI patients, and if bone marrow could correct genetic disorders of the mesenchyme (Horwitz et al. 1999). Evaluating three young children with type III OI, a single dose of unmanipulated bone marrow was infused i.v. into the patients after full myeloablative treatment (Table 3). Donor cells came from siblings and were HLA-identical or single-antigen mismatched. Only one patient demonstrated toxicity related to the transplant, and the authors stated his complications were resolved by the end of the study. Though it was not able to be determined in one patient, the other two showed engraftment, albeit at low levels (≤2%), of donor cells that were functional for at least 6 months. In all three patients, bone histology and mineralization improved, most likely due to the engraftment of donor cells. Bone biopsies before treatment showed the characteristic “woven bone” of OI, high bone turnover, poor mineralization, and the disorganized formation of new bone. However, after treatment, there were less osteocytes, more osteoblasts, organized osteoblasts, and a substantial increase in mineralization. Interestingly, the accumulation of bone mineral did not increase weight gains or body length as expected. Results were similar between all three patients. With the improved architecture and mineralization of bone, clinical improvement was seen with a drastic reduction in the number of fractures and median growth velocity increased to predicted median values. This early study showed that allogenic cells could engraft, and that low engraftment could still lead to increased mineralization, improved bone histology, and decreased fractures.

**Table 3:** Clinical studies of MSCs in the treatment of OI

Type of OI	Cases	Treatment	Methods	Results	References
III	3	Unmanipulated BM, HLA-identical or single-antigen mismatched siblings	IV, single infusion, moderate-dose total-body irradiation in mismatched donor	• Osteocytes decreased, osteoblasts increased • Increase in bone mineralization • Normalization of bone remodeling • Reduced fractures • ≤ 2% engraftment • Accelerated linear growth, but transient • 2/3 showed no toxicity	Womack 2014
				• This is an extended follow-up of the above study • This study included 2 control patients • Evaluate patients up until 4 years of age • Growth rate immediately after treatment slowed, but mineralization increased • Decreased incidence of fractures remained	Yi et al. 2020
III	6	Allogeneic BMSCs, Siblings or unrelated donors	IV, two infusions	• 5/6 patients showed engraftment of BMSCs @ 6 months • Engrafted in multiple tissues • Increased growth velocity @ 6 months • 1/6 had increased bone mineralization • No significant toxicity	Yu et al. 2021
III	1	Allogeneic HLA-mismateched FL-MSCs, 10-week male fetus Bisphosphonate treatments beginning at 4 months old	IUT 32 weeks gestation	• Engraftment was 7% at 9 months of age • Donor cells differentiated into bone cells • Mature trabecular • Minimal fractures within 2 years of age • Growth normal for child’s growth curve at 2 years of age • IUT of FL-MSCs was safe	Zhang et al. 2015
		Booster from same FL-MSC donor	IV at 8 years old	• After 2 yrs. of age, growth rate decreased and fracture rate increased • Scoliosis developed • Donor cells not found in tissue @ 6 years of age • 9 months after booster, low engraftment levels found in bone • No fractures for two years after booster, growth velocity resumed • Ability to walk and participate in sports improved	Zhou et al. 2008
IV	1	Allogeneic HLA-mismateched FL-MSCs, 7-week male fetus	dose 1: IUT at 31 weeks, dose 2: IV at 19 months	• Low engraftment levels • Growth velocity plateaued ~ 12 months of age • Growth velocity increased after both injections	Zhou et al. 2008; Zhukareva et al. 2010
III and IV	2	HLA-haploidentical BMSCs from healthy siblings	5 infusions, each 5-6 mo apart	**TERCELOI Clinical Trial** • No adverse effects with 5 treatments • Increased bone mineralization, trabecular thickness remained constant • Improved bone microstructure, but transient in severe patient • Reduced fractures • Treatment upregulated in sera: ECM, collagen binding, oxidoreductase activity, unsaturated fatty acid biosynthesis, osteogenic transcription factors • In sera, treatment downregulated: hypoxia, angiogenesis, collagen metabolic process, pro-adipogenic transcription factor	Yüksel Ülker et al. 2021
III	1 (ongoing)	HLA-mismatched FL-MSCs	IV and IO	**BOOST2B Clinical Trial** (ongoing as of this publication) • Increase in growth velocity	Lazennec andet al. Jorgensen 2008
III, IV	ongoing	HLA-mismatched FL-MSCs	IUT and IV	**BOOSTB4 Clinical Trial** (ongoing)	Zhytnik et al. 2020

*Abbreviations*: *mo* month / months, *IO* Intraosseous, *IV* Intravenous, *IP* Intraperitoneal, *IUT* In utero transplantation, *ECM* Extracellular matrix

A follow-up study evaluated the longer term clinical effects of bone marrow transplant on the type III OI children from the previous study (Horwitz et al. 1999; Horwitz et al. 2001). This longer term study of these three patients was also able to compare results against two control patients. The increase in median growth velocity seen in the immediate months after transplantation slowed down, but still did increase some. Despite the slowed growth velocity, bone mineralization still increased showing that growth and mineralization are two separate events. The decrease in fractures seen in the months after transplantation continued to remain reduced.

A follow-up study evaluated if: 1) allogenic BMSCs could engraft and have a therapeutic effect without irradiation of the patient, 2) if allogenic BMCs could engraft in multiple tissues, and 3) if two doses of BMSCs would show toxicity or increase clinical benefits (Horwitz et al. 2002). Six children with type III OI and who had received a bone marrow transplant, were given two separate i.v. injections of BMSCs that were transfected with neo^R^ gene. The BMSCs were from the original bone marrow donors, which consisted of 4 siblings and 2 unrelated donors. BMSCs used for the first injection were minimally cultured and injected at 10^6^ cell/kg. The timing of the first injection ranged from 18 to 34 months after the bone marrow transplant. The second dose consisted of culture-expanded BMSCs and was administered 8-21 days after the first dose. Though investigators aimed for a concentration of 5 × 10^6^ cell/kg, it ranged from 1-5 × 10^6^ cell/kg due to dissimilar growth patterns of BMSC donors. Engraftment at 6 weeks after the last BMSC infusion was seen in 5 out of 6 patients, occurred with both minimally cultured or expanded BMSCs, presented in different tissue types, and did not exceed 1%. Only one patient had a substantial increase in bone mineralization, however, five out of six patients had a significant increase in growth velocity. The patient that did not show an increase in growth velocity was also the only patient to show antibodies to fetal bovine serum after the second infusion, as well as the lack of detection of any donor cells in any tissue. The expression of a foreign protein, such as neo^R^, may cause the immune system of some patients to attack transduced cells. The results from this study showed that: 1) allogenic BMSCs can engraft in multiple tissues without irradiation, and 2) there are distinct mechanisms for bone mineralization and bone growth. The results of the study concluded that the BMSCs engrafted in the patient’s defective bone, where they differentiated into osteoblasts and extended the benefits of the bone marrow transplant (BMT).

The goal of the Mesenchymal Stem Cell Therapy for the Treatment of Osteogenesis Imperfecta (TERCELOI; ClinicalTrials.gov ID: NCT02172885) clinical trial was two-fold: 1) to evaluate the safety and efficacy of multiple BMSC injections, and 2) to elucidate the mechanism of action of BMSC therapy in OI patients (Infante et al. 2021). Infante et al. reported results from two patients who received a series of 5 injections of HLA-histocompatible sibling donor cells. One patient was 6 years of age at the beginning of treatment and diagnosed with type III OI while the other was 8 years of age and diagnosed with type IV OI. Both patients received 5 infusions, with each infusion 5-6 months apart, and then they were monitored and evaluated for 2 years after the last infusion. Neither patient had any adverse reactions to repeated cell therapy injections. Additionally, patients had reduced fractures and increased bone mineralization during treatment and follow-up; improved bone microstructure, although in the severe OI patient, it returned to pretreatment levels by the end of the follow-up period; the trabecular thickness remained constant; and the trabecular separation decreased in the severe patient (Ramesh et al. 2021; Infante et al. 2021). It is worth noting that Ramesh et al. points out that the TERCELOI trial uses a technique to evaluate trabecular bone microstructure that is not validated or ideal for pediatric radiographs of lower femurs (Ramesh et al. 2021).

Infante et al. took sera samples from both patients before and after treatment for mechanistic studies (Infante et al. 2021). They evaluated levels of proteins, genes, miRNAs, and transcription factors. Proteins, genes, and transcription factors for angiogenesis and adipogenesis were downregulated or inhibited. Fracture-associated miRNAs were also decreased. Increased were proteins and genetic information for cell migration, cell survival, osteogenesis, and collagen binding. Altogether, this shows that BMSC treatment has a systemic, early-osteogenic effect in OI patients via paracrine activities. Furthermore, the most severely affected patient had the most robust results with BMSC therapy as well as the most robust results with molecular findings. This suggests the clinical improvements are a consequence of the BMSC treatment, and the effectiveness of the BMSC treatment may depend on the severity of OI disease.

### FL-MSCs

To combat the low engraftment and transient therapeutic effects from previous studies, Le Blanc et al. used MSCs derived from fetal livers (FL-MSCs). FL-MSCs were shown to expand at a faster rate than BMSCs and would, therefore, not need to have prolonged time in culture, which may increase their engraftment and differentiation (Le Blanc et al. 2005). They tested the hypothesis that fetal cells would survive better than adult cells in a fetal environment, so they isolated FL-MSCs from a 10-week old male fetus and expanded them until passage 2. Cells were injected through the umbilical vein at 32 weeks and demonstrated a safe transplant. At 9 months of age, a biopsy showed ~ 7% engraftment of donor cells. Donor cells had differentiated into bone cells and showed evidence that they provided a continuous source of osteoblastic progenitors. Bone morphology looked normal, and signs of healing were not apparent, indicating that undiagnosed fractures did not occur. From the IUT transplant until the bone biopsy at 9 months, the child grew at a discernable rate. Though the child was administered bisphosonate at 4 months due to osteopenia, it would not account for the growth. Thus, the investigators surmised it must be the FL-MSCs. By age of 2 years, fractures were minimal, children had a normal growth velocity for their own growth curve, and there were no hospitalizations.

This patient was monitored for the next few years. Between the ages of 2-8 years old, her growth rate decreased, fracture rate increased, and scoliosis developed (Götherström et al., 2014). Additionally, biopsies of multiple tissues at the age of 6 found no trace of donor cells. For these reasons, as well as a lack of patient antibodies towards MSCs or FBS, a second injection of FL-MSCs was given to the patient at the age of eight. The FL-MSCs came from the same donor and did not cause a reaction when used for a second time. After the FL-MSC injection, there were no fractures reported for 2 years, and the growth velocity resumed to normal levels. The patient’s ability to walk improved, and she was able to participate in more sports. A bone biopsy from 9 months after the FL-MSC booster showed engraftment, albeit very low levels.

A patient with type IV OI was also treated with FL-MSCs pre- and postnatally (Götherström et al, 2014; Sagar et al. 2018). In utero, fresh and healing fractures were identified along with short long bones. At 31 weeks of gestation, the fetus was transplanted with FL-MSCs. For the rest of the pregnancy or infancy the baby did not present with any fractures, and at 1 month of age the patient was started on bisphosphonate therapy. Similar to the patient with type III OI, the IUT of FL-MSCs appeared to have a transient effect on the patient with type IV OI. At 1 year of age, her longitudinal length plateaued. Therefore, a second injection from the same FL-MSC donor was given at 19 months of age. No alloreactivity or adverse reactions occurred, her growth velocity resumed to normal rates, and she started to walk at a normal milestone of childhood development.

There are currently two different ongoing multi-center clinical studies evaluating HLA-mismatched FL-MSCs for OI. The Boost to Brittle Bones (BOOST2B; ClinicalTrials.gov ID: NCT04623606) clinical trial is evaluating the safety and tolerability of repeated i.v. and i.o. infusions on children with severe OI from 1 to 4 years of age (Ramesh et al. 2021; Madhuri et al. 2021). Donor cells will be expanded in culture and administered in four doses at 4 month intervals. Patients will be evaluated at 16 months after the 1st infusion and evaluated for timing of fractures, fracture frequency, bone mineralization, growth, clinical status, and bone turnover. The Boost Brittle Bones Before Birth (BOOSTB4; ClinicalTrials.gov ID: NCT03706482) clinical trial will also evaluate culture expanded FL-MSCs, but during the prenatal and infancy period (Sagar et al. 2018). Focused on severe but not lethal forms of OI, this study is intended to evaluate the safety, effectiveness, cost, and public acceptability. The investigators have already reported that major stakeholders, including OI families, OI clinicians, and patient advocate groups, have a general positive view of using FL-MSCs during the prenatal period. Though there were concerns with the origins of FL-MSCs, the safety and efficacy, and the potential side effects of the treatment, most surveyed considered early treatment advantageous to decrease the severity of OI, prevent fractures, and provide psychological benefits (Hill et al. 2019).

## Conclusions

The hope of cell therapy is that it extends beyond a temporary clinical improvement and corrects the underlying cellular and molecular defect. MSCs have shown promise in preclinical and clinical studies of OI. Based on the above studies, MSCs from different sources share six notable features. First, they are generally safe with little to no reported signs of adverse reactions. They can be safely administered prenatally, postnatally, with multiple administrations, and through different routes. They show little signs of alloreactivity and can even be HLA-mismatched. Second, they are altered in diseased states such as OI. BMSCs and ASCs, from both OI mice and human patients, had a reduced osteogenic potential, impaired mineralization ability, produced an immature ECM, and were less therapeutic in OI models. Third, they migrate to fracture sites and the growth plate of long bones. Four, they are only able to engraft at low levels of 0-7%. Engraftment can be increased with high dose radiation or priming with chemokines. Fifth, they provide a transient improvement in clinical outcome. Boosters may be required for some patients. Lastly, the therapeutic effectiveness of MSCs may be due, at least in part, to their paracrine activities.

## Data Availability

Not applicable.

## Abbreviations

AD: Autosomal dominant
ALP: Alkaline phosphatase
AR: Autosomal recessive
ASCs: Adipose derived MSCs
BMSCs: Bone marrow derived MSCs
BMT: Bone marrow transplant
BOOST2B: Boost to Brittle Bones clinical trial
BOOSTB4: Boost Brittle Bones Before Birth clinical trial
CFU: Colony forming unit
CSC: Early chorionic stem cells
DI: Dentinogenesis imperfecta
ECM: Extracellular matrix
EVs: Extracellular vesicles
FL-MSCs: Fetal liver derived MSCs
fMSCs: Fetal MSCs from peripheral blood
GMP: Good Manufacturing Practice
HPCs: Hyperplastic calluses
IO: Intraosseous
IP: Intraperitoneal
ISCT: International Society for Cell Therapy
IUT: Intrauterine transplant
IV: Intravenous
MSCs: Mesenchymal stem cells, medicinal signaling cells, marrow stromal cells
OOA: Old Order Amish
OI: Osteogenesis imperfecta
PBS: Phosphate buffer saline
SD: Sub-dermal
SDF: Stromal derived factor
SVF: Stromal vascular fraction
TERCELOI: Treatment of Osteogenesis Imperfecta clinical trial
WT: Wild type
